# Magnetic Nanotag-Based Colorimetric/SERS Dual-Readout Immunochromatography for Ultrasensitive Detection of Clenbuterol Hydrochloride and Ractopamine in Food Samples

**DOI:** 10.3390/bios12090709

**Published:** 2022-09-01

**Authors:** Ting Wu, Jiaxuan Li, Shuai Zheng, Qing Yu, Kezong Qi, Ying Shao, Chongwen Wang, Jian Tu, Rui Xiao

**Affiliations:** 1 Anhui Province Key Laboratory of Veterinary Pathobiology and Disease Control, College of Animal Science and Technology, Anhui Agricultural University, Hefei 230036, China; 2 Beijing Institute of Microbiology and Epidemiology, Beijing 100850, China; 3 College of Life Sciences, Anhui Agricultural University, Hefei 230036, China

**Keywords:** immunochromatographic assay, magnetic SERS tags, dual-readout, β2-adrenoceptor agonists, simultaneous detection

## Abstract

Direct and sensitive detection of multiple illegal additives in complex food samples is still a challenge in on-site detection. In this study, an ultrasensitive immunochromatographic assay (ICA) using magnetic Fe_3_O_4_@Au nanotags as a capture/detection difunctional tool was developed for the direct detection of β2-adrenoceptor agonists in real samples. The Fe_3_O_4_@Au tag is composed of a large magnetic core (~160 nm), a rough Au nanoshell, dense surface-modified Raman molecules, and antibodies, which cannot only effectively enrich targets from complex solutions to reduce the matrix effects of food samples and improve detection sensitivity, but also provide strong colorimetric/surface-enhanced Raman scattering (SERS) dual signals for ICA testing. The dual readout signals of the proposed ICA can meet the detection requirements in different environments. Specifically, the colorimetric signal allows for rapid visual detection of the analyte, and the SERS signal is used for the sensitive and quantitative detection modes. The proposed dual-signal ICA can achieve the simultaneous determination of two illegal additives, namely, clenbuterol hydrochloride and ractopamine. The detection limits for the two targets via colorimetric and SERS signals were down to ng mL^−1^ and pg mL^−1^ levels, respectively. Moreover, the proposed assay has demonstrated high accuracy and stability in real food samples.

## 1. Introduction

β2-Adrenoceptor agonists, such as clenbuterol hydrochloride (CLE) and ractopamine (RAC), can promote fat degradation and protein synthesis; as such, they are often illegally used as lean meat extracts in animal husbandry to improve lean meat yield [1]. However, CLE and RAC are not easily metabolized in animals, and long-term human intake can seriously affect health [2]. Various countries have strict limits or outright bans on these β2-adrenoceptor agonist residues in animal food [3]. Laboratory detection techniques, including high-performance liquid chromatography (HPLC) [4], liquid chromatography–mass spectrometry [5], and gas chromatography [6], have been applied to accurately analyze β2-adrenoceptor agonists. However, these methods require skilled personnel, sophisticated instruments, and specialized laboratory rooms, and are thus unsuitable for on-site detection. An effective point-of-care testing (POCT) method is important for rapid, direct, and real-time monitoring of hazardous additives in foods, but it has yet to be developed.

Immunochromatographic assay (ICA), one of the most promising POCT techniques, has been widely used in the on-site detection of various analytes due to its advantages of simple operation, short detection time, low cost, and suitability for mass production [7,8,9]. For small molecules (e.g., antibiotics, metal ions, and mycotoxins) [10,11] in particular, the ICA method is constructed by an indirect competitive immunoassay, which can effectively avoid false positives at high concentrations of analytes [12,13]. However, traditional ICA methods, which typically utilize colloidal gold (AuNP) to provide visual colorimetric signals, have inherent defects and are of limited sensitivity and quantitative capacity. To overcome these defects, in recent years, several dual-signal nanotags (e.g., SiO_2_@Au/quantum dot, AuNP-HRP, and Au nanostar) have been introduced into the ICA system to replace the traditional AuNP tags, and have retain the stable colorimetric signal for rapid detection of the target and provide a more sensitive and quantifiable signal (fluorescent, Raman, and chemiluminescence) for accurate analysis [14,15,16]. These dual-signal ICA methods exhibited higher sensitivity, wider detection range, and more flexible application scenarios [17,18]. Surface-enhanced Raman scattering (SERS), a Raman signal amplification phenomenon generated on the surface of “free-electron-like” metal materials (e.g., Au, Ag), is a powerful, fingerprint-specific vibrational spectroscopy technique [19,20,21]. The SERS labeling method has distinct advantages, including high sensitivity (single molecule level), high stability (no photobleaching), and fingerprint identification spectrogram, thus attracting great attention [22,23,24]. Researchers have achieved the quantitative analysis of various biomolecules (e.g., protein toxin, biomarkers, nucleinic acid, virus, and immunoglobulins) with high sensitivity by utilizing SERS signal-based ICA methods [25,26,27,28,29]. However, due to the complex matrixes (e.g., proteins, salts, and acid environment) of actual food samples that can easily affect the colloidal stability of common SERS nanotags, a SERS-ICA method for the simultaneous detection of illegal additives in food has not been reported yet.

Owing to its excellent properties, which include high stability, biocompatibility, and magnetic responsiveness, magnetic nanoparticle (MNP) has been widely used to construct stable biosensors for the direct detection of analytes in complex environments, such as blood, urine, sputum, river water, and various food samples [30,31,32]. Moreover, the unique magnetic response property of MNP allows for the rapid separation and purification of target analytes from complex samples, which can effectively avoid matrix interference, improve detection sensitivity, and save time by replacing complicated sample pretreatment steps such as extraction, purification, washing, and centrifugation [33,34,35]. Recently, the combination of magnetic nanocomposites (e.g., magnetic SERS materials, magnetic fluorescent microspheres, and magnetic nano-enzymes) and the ICA system has enabled accurate and sensitive detection of biomacromolecules (mainly pathogens and biomarkers) in complex samples, thus indicating the tremendous potential of magnetic tags-based ICA methods in on-site detection [36,37,38].

Inspired by these aforementioned works, herein, we reported a colorimetric/SERS dual-readout ICA biosensor based on high-performance Fe_3_O_4_@Au (Mag@Au) as the magnetic enrichment/SERS detection difunctional tool for the ultrasensitive and simultaneous detection of two β2-adrenoceptor agonists in food samples. The proposed assay has the following three advantages compared to the previously reported ICA biosensors for β2-adrenoceptor agonists: (i) Mag@Au SERS nanotags were introduced to the ICA method to capture the target CLE/RAC and enrich them through the magnetic field, which can remove the interference and greatly improve the sensitivity. To the best of our knowledge, this study is the first to propose a magnetic SERS tag-based competitive ICA method and achieve the simultaneous analysis of two small molecules on a magnetic-based ICA strip. (ii) The excellent performance (superior SERS activity, good stability, and good dispersion in complex samples) of the proposed Mag@Au tags enables the direct and simultaneous detection of two β2-adrenoceptor agonists in one ICA strip with ultra-high sensitivity (pg mL^−1^ level by SERS signal). (iii) The Mag@Au-based ICA can provide a colorimetric/SERS dual-signal readout, which can meet the needs of different testing environments and improve the detection flexibility of the method. Under optimal conditions, the cut-off values of our method, based on the colorimetric signal for CLE and RAC detection, were 1 and 0.33 ng mL^−1^, respectively. Both values were 10 times more sensitive than the traditional AuNP-based ICA method. The limits of detection (LODs) for CLE and RAC by SERS signal were 7.8 and 3.5 pg mL^−1^, respectively, which were 115 and 217 times higher than those of AuNP nanotag-based SERS-ICA. Moreover, we have also validated the accuracy, stability, and specificity of the proposed method in real samples (pork, beef, and mutton). We believe that the proposed colorimetric/SERS dual-readout ICA has great potential for POCT use.

## 2. Experimental Section

### 2.1. Chemicals, Materials and Instruments

Clenbuterol hydrochloride (CLE) and ractopamine (RAC) were purchased from Yuanye Bio-Technology Co., Ltd. (Shanghai, China). Anti-CLE monoclonal antibodies (catalog no. QY-C0109), anti-RAC monoclonal antibodies (catalog no. QY-30004), coating antigen CLE-BSA (catalog no. QYL-040101), and coating antigen RAC-BSA (catalog no. QYL-040106) were acquired from Qiyi Biotechnology Co., Ltd. (Shanghai, China). Polyclonal goat anti-mouse IgG antibody was purchased from Sangon Biotech Co., Ltd. (Shanghai, China). Branched polyethyleneimine (PEI, MW 25 kDa), polyvinylpyrrolidone (PVP, 40 kDa), 5, 5′-dithiobis-(2-nitrobenzoic acid) (DTNB), tween-20, 1-(3-dimethylaminopropyl)-3-ethylcarbodiimide hydro (EDC), N-hydroxysuccinimide (NHS), 2-(N-morpholino) ethanesulfonic acid (MES), bovine serum albumin (BSA), and sodium azide (NaN_3_) were obtained from Sigma–Aldrich (St. Louis, MO, USA). Phosphate-buffered saline (PBS, pH = 7.4), chloroauric acid tetrahydrate (HAuCl_4_·4H_2_O), trisodium citrate (TSC), and hydroxylamine hydrochloride (NH_2_OH·HCl) were provided from Sinopharm Chemical Reagent Co. (Shanghai, China). Nitrocellulose (NC) membranes (Sartorious CN140 with 8 µm pore size) were bought from Sartorius (Gottingen, Germany). Absorbent pads, sample pads, and polyvinyl chloride (PVC) plates were supplied from Jieyi Biotechnology Co., Ltd. (Shanghai, China).

Transmission electron microscopy (TEM) images were performed with the Tecnai G2 F20 microscope (Philips, Amsterdam, Holland). Scanning electron microscopy (SEM) images were taken with a JSM-7001F microscope (JEOL, Tokyo, Japan). The zeta potentials were measured with a Malvern Nano-ZS90 Zetasizer. All Raman spectra of SERS-based ICA strips were acquired with a B&W Tek, i-Raman Plus BWS465–785H spectrometer. The acquisition time for Raman detection is typically 10 s. The sample excitation is performed using a 785 nm laser with a power of 10 mW. The SERS mapping images of the test lines were scanned by the Renishaw invia plus Raman system to evaluate the homogeneity of the SERS signal. Incident radiation was coupled to an Olympus BX51 optical microscope using a computer-controlled x-y panning stage to scan a 30 × 30 μm T-zone over 750-μm (*x*-axis) and 600-μm (*y*-axis) (500 pixels total). The laser power was 1%, the acquisition time was 1 s, and the SERS datas were processed by Renishaw Wire 4.2 software.

### 2.2. Synthesis of Mag@Au NPs

**Scheme 1:**
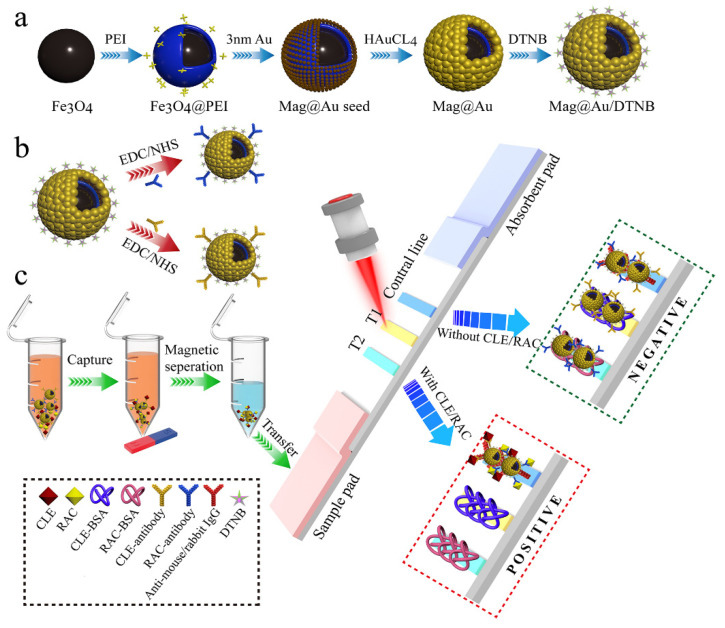
Schematic of (**a**) the synthesis process of Mag@Au/DTNB NPs, (**b**) the process of Mag@Au/DTNB NPs coupled with antibodies, and (**c**) the detection principle based on dual-readout Mag@Au-ICA for the simultaneous detection of CLE and RAC.

High-performance gold-coated magnetic nanoparticles were synthesized as shown in Scheme 1a. First, the magnetic cores (~160 nm Fe_3_O_4_ MNPs) and small Au seed NPs (~3 nm) were fabricated by the previously reported solvothermal method [39] and NaBH_4_ reduction method [40], respectively. Then, the prepared Fe_3_O_4_ MNPs (1 mg mL^−1^) were uniformly dispersed into 40 mL of PEI solution (1.0 mg mL^−1^) and treated under sonication for 15 min to ensure that the PEI was wrapped around the magnetic core [41]. After rinsing twice with deionized water to remove the excess PEI, the Fe_3_O_4_@PEI MNPs were mixed into 50 mL of AuNPs (3 nm) solution and the mixture was sonicated for 30 min to obtain magnetic Fe_3_O_4_@Au (Mag@Au) seed nanoparticles. Subsequently, the solution of Mag@Au seed NPs was ultrasonically dispersed into 40 mL of the deionized aqueous solution containing NH_2_OH·HCl (1.0 mg mL^−1^) and PVP (1.2 mg mL^−1^). Then, a certain amount of HAuCl_4_·4H_2_O (40 μM) was added. After sonicating for 15min, the Au shell was completely formed through a redox reaction. Finally, the prepared Mag@Au was rinsed twice with deionized water and stored in ethanol for later use.

### 2.3. Preparation of Antibody-Conjugated Mag@Au Tags

The preparation process of Mag@Au tags is divided into two main steps. First, the carboxyl-containing Raman reporter molecule (DTNB) was modified onto Mag@Au NPs. Then, the specific antibody was coupled to the nanomaterial by the diimine carbon method. Specifically, 1 mL of Mag@Au NPs was mixed with 10 μL of DTNB (10 mM). After sonication for 1 h, Mag@Au/DTNB NP was formed and rinsed twice with ethanol. Subsequently, 1 mL of Mag@Au/DTNB NPs was resuspended into the MES buffer (pH 5.5, 10 mM) and mixed with 5 μL EDC (100 mM) and 10 μL NHS (100 mM). The mixture was sonicated for 15 min, and the excess EDC and NHS were removed by magnetic separation. Thereafter, 8 μg of the capture antibody was added to the activated nanomaterial and incubated for 2 h. Subsequently, 80 μL of 10% BSA (*w/v*) was added and incubated for 1 h to block unreacted residual binding sites. After rinsing with 0.05% PBST and magnetic collection, the Mag@Au SERS tags were dissolved into a 0.05% PBST solution containing 0.02% NaN_3_ (*w/w*) and stored at 4 °C.

### 2.4. Manufacturing of the ICA Strip

The ICA strip was composed of four parts: an absorbent pad, an NC membrane, a sample pad, and a PVC plate. The NC membrane was attached to the PVC plate before the goat anti-mouse antibody (0.8 mg mL^−1^), RAC-BSA (mg mL^−1^), and CLE-BSA (0.06 mg mL^−1^) were uniformly sprayed onto the NC membrane as the C-line, T1-line, and T2-line, respectively. Then, the NC membrane was dried at 37 °C for 2 h. The absorption and the sample pads were cut into appropriate sizes and superimposed on both ends of the NC membrane with an overlap of about 2 mm between the adjacent parts. Finally, the assembled ICA strips were cut into 3 mm width and put into the vacuum dryer for long-term storage.

### 2.5. Simultaneous Detection of CLE and RAC via Dual-Readout Mag@Au-ICA

To evaluate the performance and sensitivity of the proposed assay, a series of mixed standard solutions containing different concentrations of CLE (0–3 ng mL^−1^) and RAC (0–3 ng mL^−1^) were detected by the Mag@Au-ICA system under optimized parameter conditions. Approximately 3.5 μL of the functionalized Mag@Au SERS tags was mixed with 1 mL of the standard detection solution. After 5 min of incubation, the supernatant was removed by magnetic enrichment and 80 μL running buffer (10 mM PBS, 1% Tween) was added. The previously prepared ICA strips were used for detection. The SERS signals of the two T-lines were measured and analyzed with a portable Raman instrument (the excitation power is 10 mW, and the acquisition time is 10 s). Finally, semi-quantitative and quantitative analyses of CLE and RAC were carried out based on the intensity of the collected colorimetric and SERS signals.

### 2.6. Detection of RAC and CLE in Real Samples

To verify the reliability of the dual-readout Mag@Au-ICA in practical applications, pork, beef, and mutton were selected as actual samples, and the performance of the Mag@Au-ICA was analyzed by the standard addition method. Five grams of pork, beef, and mutton samples were pounded and dispersed into 10 mL of deionized water. Then, the mixture was sonicated (10 min) to ensure adequate extraction of CLE and RAC. The supernatant was then extracted by filtration and centrifugation (10,000 rpm, 10 min). Known concentrations of CLE and RAC standards were added to the sample solution, and the Mag@Au-ICA was operated according to the established conditions. The assay was repeated five times for each sample. Finally, the measured values were substituted into the established calibration curves to calculate the recoveries of CLE and RAC in the food samples.

## 3. Results and Discussion

### 3.1. Strategy for the Dual-Readout Mag@Au-ICA Based on Mag@Au Tag

The Mag@Au tag was used as a multifunctional capture/detection tool to construct the dual-readout ICA biosensor, which is the key to achieving fast enrichment and highly sensitive detection of the two β2-adrenergic agonists in the food samples. As shown in Scheme 1a, the Mag@Au tag consists of a 160 nm Fe_3_O_4_ MNP as the superparamagnetic core and a rough Au nanoshell to provide high SERS activity and surface sites for 5, 5′-dithiobis-(2-nitrobenzoic acid) (DTNB) modification. The carboxyl groups of the surface-modified DTNB can be used for direct conjugation of antibodies (Scheme 1b). The prepared Mag@Au tags can effectively capture the target analytes from the sample solution, thus simplifying the complex pretreatment process, reducing matrix effects, and improving the sensitivity of ICA method. Moreover, the micro-size (200 nm) effect of Mag@Au can also enhance colorimetric ability, thus generating a more sensitive visual signal on the ICA strip [42].

Scheme 1c demonstrates the sequential steps of Mag@Au-ICA for the ultrasensitive detection of the two β2-adrenergic agonists, which are based on a typical competitive immunoreaction between CLE/RAC in the sample and two haptens (CLE-BSA, RAC-BSA) immobilized onto the test lines of the NC membrane for binding with immuno-Mag@Au tags. The detection process can be divided into two parts: first, the Mag@Au tags were added to the sample solution to quickly capture the targets and enrich them by magnetic force; second, the Mag@Au-target complexes were dispersed in the running solution and then loaded onto the sample pad of the test strip to start the immunochromatographic reaction. Based on the competition principle, the target analyte in the sample solution competes with the antigen immobilized on the T-line to bind the Mag@Au tags. The more target analytes in the sample solution, the more specific the binding sites of the antibody are occupied; the antigen on the corresponding T-line will not bind to the Mag@Au tag. Furthermore, color band is always present in the C-line region to verify the validity of ICA. Finally, the corresponding qualitative or quantitative analyses are carried out according to colorimetric or SERS signal. Notably, 785 nm excitation wavelength was chosen for the proposed Mag@Au-ICA method due to its ability to effectively reduce the autofluorescent background on the ICA strip [43].

### 3.2. Characterization of Mag@Au Tags

**Figure 1:**
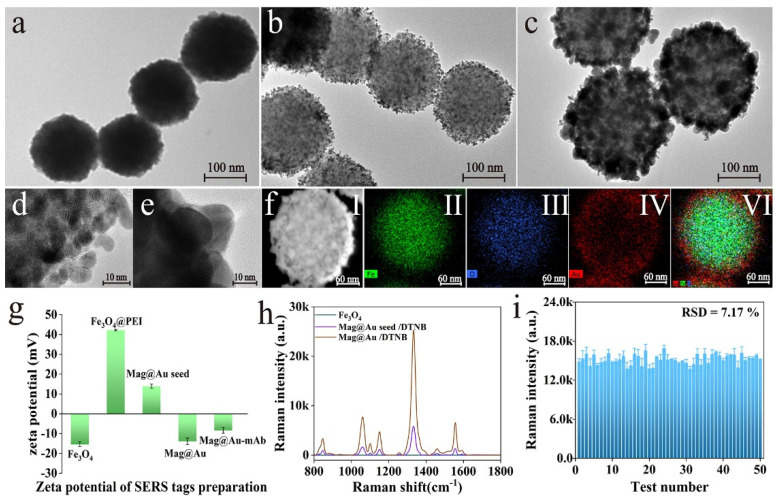
Characterization of synthetic Mag@Au tags. TEM images of (**a**) Fe_3_O_4_ MNPs, (**b**) Mag@Au seed NPs, and (**c**) Mag@Au NPs. HRTEM images of (**d**,**e**) single Mag@Au seed NP and I Mag@Au NP at magnification. (**f**) Elemental mapping images of single Mag@Au NPs. (**g**) Zeta potential map. (**h**) SERS signal map. (**i**) Raman signal intensity at 1331 cm^−1^ of different batches of Mag@Au tags, which was measured 50 times.

The size and morphology of the magnetic particles obtained in the synthesis process were characterized in detail by transmission electron microscopy (TEM). Figure 1a–c show the TEM images of Fe_3_O_4_ MNPs, Mag@Au seed NPs, and Mag@Au NPs, respectively. The Fe_3_O_4_ MNPs with a uniform particle size of ~160 nm were prepared by an improved solvothermal method (Figure 1a). After wrapping Branched polyethyleneimine (PEI), AuNPs (3 nm) were absorbed by electrostatic adsorption (Figure 1b). Then, the reduced gold atoms were deposited on the Au seeds on the surface of the magnetic core to form an Au shell under the action of ultrasonic waves, with NH_2_OH•HCl as a reducing agent and HAuCl_4_·4H_2_O as the gold precursor (Figure 1c). The surface of the MNPs became rougher and the particle size increased from 160 nm to 200 nm. The detailed surface morphologies of the Mag@Au seed NP and the Mag@Au NP were further studied using enlarged single-particle HRTEM images (Figure 1d,e). It can be clearly seen that 3 nm AuNPs were adsorbed on the surface of Fe_3_O_4_, and the thickness of the Au shell formed was ~10 nm. The elemental analysis of the single Mag@Au NP was carried out by energy spectrometry. Figure 1f displays the main elemental compositions of Mag@Au NP: Fe (green), O (blue), and Au (red). Figure 1f(VI) illustrates how the surface of the Fe core (green) is tightly wrapped by a layer of Au (red) shell, which constitutes a clear and complete core–shell structure. The synthesis of each product of the process of Mag@Au NP fabrication was monitored by measuring the zeta potential. As shown in Figure 1g, the zeta potential of the pure magnetic particle is −15.4 mV, which increased to +42.2 mV after wrapping the PEI. The large amount of ammonium roots carried by PEI makes the nanoparticle surface carry a large positive charge, which results in a positive surface potential of the magnetic nucleus. After adsorption of the negatively charged 3 nm AuNPs and the synthesis of Au shells, the potential kept decreasing to −17.6 mV. Meanwhile, the ultraviolet–visible absorption spectra (UV–vis) of Mag@Au NPs were investigated. As shown in Figure S1, the magnetic core exhibits a clear resonance absorption peak at 559 nm after wrapping the Au shell. This phenomenon is due to the resonance and the coupling of homogeneous excitations on the surface of adjacent AuNPs, which redshifts the resonance absorption peak.

Subsequently, the SERS activity of the Mag@Au tags was evaluated. DTNB was selected as the Raman reporter molecule for the probe because it exhibited a strong SERS signal on the gold surface, contained disulfide bonds, and could form strong Au–S bonds with a gold surface [44]. Moreover, the terminal carboxyl group of DTNB molecule could bind to the amino group of the antibody and form a stable covalent bond through the EDC/NHS chemistry [45,46]. This strategy for antibody conjugation is more effective than other coupling methods [47]. In this work, DTNB-modified Mag@Au was prepared by a saturation modification strategy. As shown in Figure S2, the SERS intensity of 100 μM DTNB modified Mag@Au tags was strong enough and rather stable, which indicated that the surface of the Mag@Au tags was saturated with DTNB molecules. In Figure 1h, the Fe_3_O_4_ core does not have Raman signals. Mag@Au/DTNB NPs have a distinct characteristic peak at 1331 cm^−1^, with significantly higher intensity than the Mag@Au seed/DTNB NPs. The reduced Au shell nanomaterial has a rougher surface to build up hot spots and has superior SERS new energy activity. In addition, the SERS signal reproducibility of Mag@Au was also evaluated. The SERS signal was measured 50 times for different batches of Mag@Au, and the results (Figure 1i) proved to have high reproducibility (relative standard deviation of 7.17%). Approximately 100 μL of Mag@Au was mixed into distilled water with different pH values (pH 3−11) and left for 24 h. The SERS signals were measured after magnetic separation. As shown in Figure S3, the signal of Mag@Au had high stability over a wide pH range. In addition, Fourier-transform infrared spectroscopy (FTIR) was used to confirm the formation of immuno-Mag@Au tags. As shown in Figure S4, the FTIR spectrum of the immuno-Mag@Au tags has a characteristic absorption peak at 1641–1530 cm^−1^ [48], which belongs to protein amide bands I and II and indicates the successful binding of the antibody.

### 3.3. Feasibility and Optimization of Dual-Readout Mag@Au-ICA for CLE and RAC

**Figure 2:**
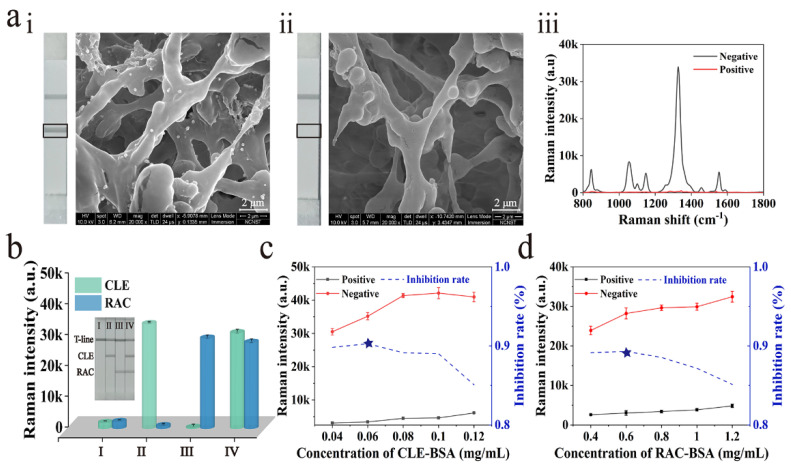
(**a**) Diagrams of the test strip and corresponding SEM images in (**i**) the negative case, (**ii**) the positive case, and (**iii**) the corresponding SERS signal intensity on the T-lines. (**b**) Graphs of the test strips with different concentrations of CLE and RAC (I: 10, 10 ng mL^−1^; II: 0, 10 ng mL^−1^; III: 10, 0 ng mL^−1^; IV: 0, 0 ng mL^−1^) and the SERS signal intensity of the corresponding T-lines. (**c**,**d**) Effect of different concentrations of CLE-BSA and RAC-BSA on the competition inhibition rate, the blue five-pointed star represents the position with the highest rate of competition suppression. The error bar is the standard deviation of five independent tests based on Mag@Au-ICA.

The T-lines on the negative and positive ICA strips were characterized by scanning electron microscopy (SEM) to verify the binding effect of Mag@Au tags and ICA strips. A large number of uniformly sized particles in tight binding can be clearly seen on the negative ICA strip (Figure 2a(i)) compared with the positive ICA strip (Figure 2a(ii)). The SERS signals in the T-line region of the negative and positive test strips were compared (Figure 2a(iii)). It was demonstrated that the SERS signals in the T-line region of the test strips originated from Mag@Au tags. Moreover, the signal difference between the negative and positive regions was large enough to be used for the quantitative analysis of the targets. The CLE and RAC (I: 10, 10 ng mL^−1^; II: 0, 10 ng mL^−1^; III: 10, 0 ng mL^−1^; IV: 0, 0 ng mL^−1^) were assayed at different concentrations according to the constructed strategy to ensure the feasibility of the multiple pathway assays of the protocol. The color band appears on the corresponding test line only when the target analyte is not present, as revealed in Figure 2b. In comparison with the blank control group, no significant difference in SERS signal intensity was observed in the two T-line regions, indicating no cross-reactivity between the two antigens.

To achieve accurate and highly sensitive detection of CLE and RAC, the key parameters affecting the experimental results, such as the pore size of the nitrocellulose (NC) membrane, the concentration of the coating antigen, incubation time, and enrichment system, were optimized. In this experiment, the competitive inhibition rate (1 − B/B_0_ × 100%) of the test system was used as the evaluation criterion to obtain the optimal detection conditions, where B and B_0_ are the SERS signals of the T-lines for the positive and negative samples of the target antigen, respectively. First, we investigated the effects of three commonly used NC membranes (prima40, CN95, and CN140) with pore sizes (18, 15, and 8 μm) on the experimental results, considering the relatively large particle size of Mag@Au tags. The results (Figure S5) based on the competitive inhibition rate showed that CN140 with 8 μm pore size exhibited better detection performance in comparison to CN95 and CN140. This phenomenon can be attributed to the CN140 membrane having a slower capillary speed than Prima40 (~18 μm pore size) and the CN95 (~15 μm pore size), which provides longer immune reaction time on the ICA strip and achieves higher sensitivity. As shown in Figure 2c,d, the SERS signal on the test line increased with the increase of semi-antigen concentration. The competitive inhibition rates were the highest (where the blue five-pointed star was located), when the concentration of RAC-BSA reached 0.6 mg mL^−1^ and the concentration of CLE-BSA reached 0.06 mg mL^−1^. In addition, the running buffer, incubation time, and reaction time during the ICA run were optimized to achieve rapid detection. The competitive inhibition rate reached its maximum at 20 min of reaction in the PBS buffer containing 1% Tween 20, as illustrated in Figure S6a,b. The inhibition effect leveled off after 5 min of incubation time, so 5 min was selected as the best reaction condition (Figure S6c). In this study, the Mag@Au tags capture and enrich the target analyte in the sample solution, causing the analyte to be pre-concentrated to a certain concentration. Theoretically, the larger the sample solution system, the higher the concentration of the pre-concentrated analytes; thus, the sensitivity of the assay is somewhat improved. However, oversized systems cause longer times and more tag loss. The effect of the enrichment system on the assay results was evaluated at a certain time (5 min) to ensure the rapidity of the assay. When the enrichment system exceeds 1 mL, the SERS signal continuously decreased with the increase in the system, as shown in Figure S6d. By comparing the inhibition rate, we chose 1 mL as the enrichment system for ICA detection with an enrichment time of 5 min, under the premise of ensuring the rapidity and simplicity of the assay.

### 3.4. Detection Performance of the Dual-Readout Mag@Au-ICA

**Figure 3:**
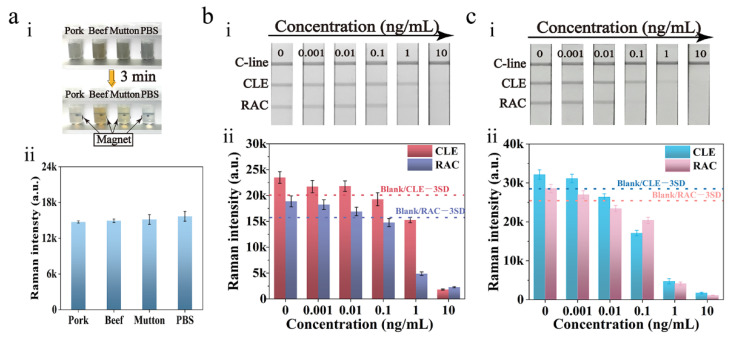
(**a**) Diagrams of (**i**) the magnetic enrichment effect of Mag@Au tags in complex samples and (**ii**) the corresponding SERS signal intensity at 1331 cm^−1^ after enrichment. The detection results of CLE and RAC are based on Mag@Au-ICA in the (**b**) unenriched and (**c**) enriched modes. Diagrams of the test strips (**i**) and the corresponding SERS signal (**ii**) in the (**b**) non-enriched mode and (**c**) enriched mode. The error bar is the standard deviation of the five independent tests based on Mag@Au-ICA.

We put the Mag@Au tags into 1 mL of the actual sample (pork, beef, and mutton) extracts to confirm the feasibility of the magnetic immuno-probe application in real complex samples (the actual samples were treated as described in Section 2.6). After magnetic enrichment for 3 min, all particles were gathered to the back of the EP tubes and the color of the sample solution returned to its own color (Figure 3a(i)). The signals of the isolated Mag@Au tags were examined, and no significant change in SERS intensity was found (Figure 3a(ii)). Such excellent properties of Mag@Au tags in complex samples can be attributed to the good biocompatibility and stability of th Fe_3_O_4_ core and Au shell. This finding indicated that Mag@Au tags can be used as an effective tool to quickly capture, concentrate, and detect CLE/RAC in real samples. Meanwhile, in order to verify that magnetic enrichment can improve the sensitivity of detection in actual samples, the multi-detection of CLE and RAC in mutton extracts was performed in enriched and unenriched modes. The comparison of the detection results in both modes (Figure 3b) demonstrates that the visualization of the combined detection and SERS sensitivity are superior in the enriched mode in comparison to the non-enriched mode. Based on the colorimetric signal, the visual limits of detection (vLODs) of 1 and 0.1 ng mL^−1^ for CLE and RAC in the enrichment mode (Figure 3c(i)) were 10 times better than those in the non-enrichment mode (Figure 3b(i)). As revealed in the Figure 3b(ii),c(ii), the limits of detection (LOD = y_blank_ − 3 × SD_blank_, where y_blank_ is the mean signal intensity of zero, and SD_blank_ is the standard deviation of blank measurement) for CLE and RAC detection in the enriched model were higher than those of the unenriched mode. Moreover, the SERS signal is stronger in the enriched mode under the same detection conditions. In the unenriched mode, impurities (proteins, fats, and other macromolecules) in the actual sample can affect the detection results. The above results demonstrate that detection by the magnetic enrichment mode has a certain improvement on the sensitivity, which is about 10 times higher.

**Figure 4:**
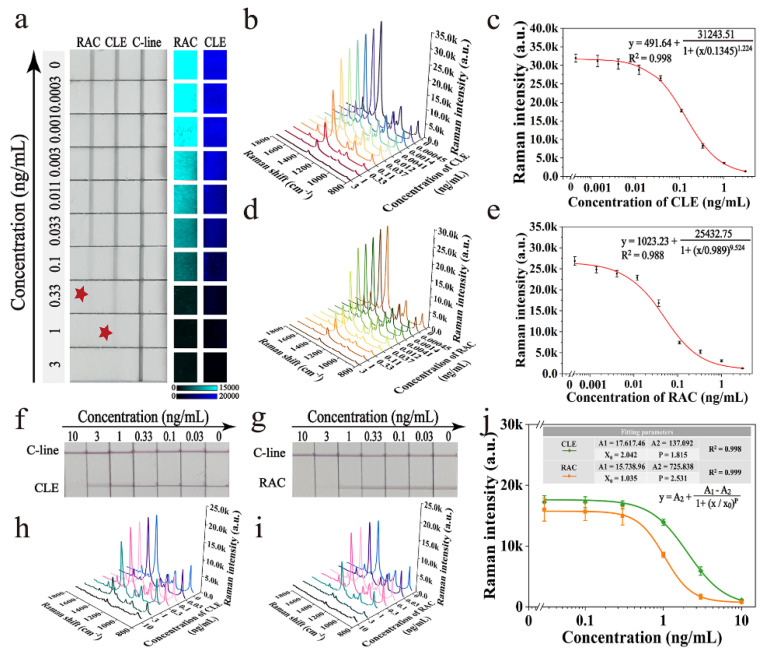
(**a**) Graphs of the test strips based on Mag@Au-ICA detection of different concentrations of CLE and RAC and the corresponding SERS mapping images. (**b**,**d**) Average SERS spectra of the two test lines and (**c**,**e**) the corresponding calibration curves. (**f**,**g**) Results of the test strips with different concentrations of CLE and RAC, (**h**,**i**) corresponding average SERS spectra, and (**j**) corresponding calibration curves based on AuNP/DTNB-ICA.

Two standard β2-adrenoceptor agonists were simultaneously detected using the established dual-readout Mag@Au-ICA biosensor under the optimized conditions to validate its multiple detection performance. Then, the sensitivity and detection range of CLE and RAC multi-detection were evaluated. Figure 4a displays the chromatographic results for the different levels of CLE and RAC in the concentration range from 0 ng mL^−1^ to 3 ng mL^−1^ and the corresponding Raman mapping images. The colors on the T-lines deepened with the decrease in the concentration of the target analyte. When the concentrations of CLE and RAC reached 1.0 and 0.33 ng mL^−1^, respectively, the colors were not visible to the naked eye. Meanwhile, 25 × 20 (1 pixel = 30 μm × 30 μm) pixels were employed to map the Raman intensity of the T-lines to monitor the homogeneity of the Raman signal of the T-lines. The darker the color of the mapping images, the lower the SERS intensity was on the test lines. Nevertheless, the mapping images are not homogeneous due to two reasons: the SERS label was attached to the front of the T-line first, and there were of different substrate conditions. To address this issue, 20 light spots are randomly measured on the T-line to eliminate the influence of the uneven SERS signals on the experimental results [26]. The Raman signals of the two T-lines in Figure 4a were read with a portable Raman spectrometer, and the corresponding SERS signal maps were obtained (Figure 4b,d). Calibration curves for CLE and RAC detection were established based on the calibrated signal intensity values at 1331 cm^−1^ (Figure 4c,e). The results indicated that the calibration curves of CLE and RAC were logarithmically related with correlation coefficients (R^2^) of 0.988 and 0.998, respectively. The LODs for CLE and RAC were calculated to be 7.8 and 3.5 pg mL^−1^, respectively. The developed magnetic SERS sensor has a relatively high sensitivity and ability of multiplex detection in comparison to the other established ICAs for the detection of β2-adrenoceptor agonists (Table 1). Theoretically, by changing the recognition element on the Mag@Au SERS tag, it is possible to detect other different types of small molecules.

We used DTNB-modified AuNP immunochromatographic test strips (AuNP/DTNB-ICA) as a control to evaluate the superiority of Mag@Au-ICA established in this experiment, and the detection results are shown in Figure 4f,g. Based on the AuNP/DTNB-ICA assay, the vLODs of CLE and RAC were 3 and 1 ng mL^−1^, and the cut-off values were 10 and 3 ng mL^−1^, respectively. The calibration curves that corresponded to CLE and RAC were constructed based on the corrected SERS signal values (Figure 4h–j). The calculated LOD values of CLE and RAC were 0.89 and 0.76 ng mL^−1^, respectively, which were much less sensitive than the Mag@Au-ICA established in this experiment. In conclusion, the sensitivity of the established Mag@Au-ICA is 10 times higher based on colorimetric signal and 115–253 times higher based on the SERS signal in comparison to AuNP/DTNB-ICA.

### 3.5. Selectivity and Stability of Mag@Au-ICA

Several common additives, such as gentamicin (GM), ofloxacin (OFLA), chloramphenicol (CAP), and salbutamol (SAL), were selected as interferents to study the specificity of Mag@Au-ICA. The four interferents were diluted into the standard solution at a final concentration of 100 ng mL^−1^, assayed with the established Mag@Au-ICA, and compared with the detection results of CLE (100 ng mL^−1^) and RAC (100 ng mL^−1^). Only the samples containing target antigens could be recognized by the immuno-label as a consequence of the ICA strips (Figure S7a), which indicates that the prepared immuno-sensor has high specificity. The SERS intensity (Figure S7b) showed that the high concentration of interfering substances had no significant effect on the detection process. A range of concentrations of CLE and RAC standard solutions (1.0, 0.1, and 0 ng mL^−1^) were detected and repeated five times under the same conditions, and the SERS signals were measured to evaluate the reproducibility of the ICA bands. The relative standard deviations (RSDs) were 8.79%, 5.06%, and 4.35% for CLE (Figure S8a) and 4.23%, 1.57%, and 9.43% for RAC (Figure S8b), respectively, at the three different concentrations (1, 0.1, and 0 ng mL^−1^). The RSDs at different concentrations are less than 10%, which indicates the reliability of the biosensor. In conclusion, the ICA test strip constructed in this experiment has good selectivity and repeatability and can be used for specific detection of CLE and RAC.

### 3.6. Application in Actual Samples

**Figure 5:**
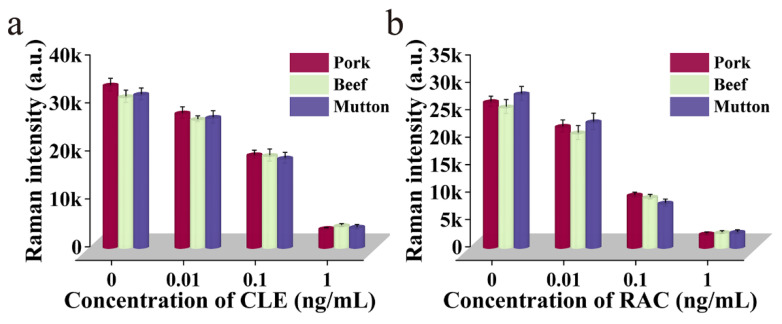
SERS signal intensity at 1331 cm^−1^ of different concentrations of (**a**) CLE and (**b**) RAC in pork, beef, and mutton extracts.

The pork, beef, and lamb were selected as the actual samples, and the standard spiked recovery test was used to evaluate the actual field detection capability of dual-readout Mag@Au-ICA. The CLE and RAC standard solutions were added to the extracts of pork, beef, and mutton, resulting in the antigenic mixture in the extracts of 1.0, 0.1, 0.01 and 0 ng mL^−1^, respectively. Then, a range of concentrations of sample solutions were assayed with Mag@Au-ICA. In Figure 5a,b, the measured signal values in the pork, beef, and mutton extracts are close to the theoretical values, which proves that the Mag@Au-ICA SERS bands are stable in different complex samples. The recoveries of the quantitative analytical results are summarized in Table S1. The recovery rate of this method in the actual sample assays ranged from approximately 89.63% to 110.6%. The study proved that certain substances, such as fat and protein, in the actual samples can interfere with detection, to a certain extent, but were within the acceptable range. In summary, the established dual-readout Mag@Au-ICA can achieve the simultaneous detection of CLE and RAC, which is vital for monitoring the illegal addition of β2-adrenoceptor agonists in the market.

## 4. Conclusions

In this study, a colorimetric/SERS dual-signal ICA biosensor was constructed for the ultrasensitive and simultaneous detection of two β2-adrenoceptor agonists (i.e., CLE and RAC) based on the multifunctional Mag@Au nanotags. The fabricated Mag@Au tag consists of a Mag@Au core, dense surface-modified Raman reporter molecules, and specific antibodies, which can be used as a capture/magnetic separation tool and output colorimetric and SERS signals for ICA detection. More importantly, Mag@Au tags can improve the sensitivity of the ICA method (10-fold) by enriching the target, and exhibit excellent stability in different food environments. Under the optimal parameter conditions, the detection limits of CLE and RAC based on the SERS signal can reach 7.8 and 3.5 pg mL^−1^, respectively. The detection limits based on the colorimetric signal were 1.0 and 0.33 ng mL^−1^, respectively. Good recoveries (89.63–110.6%) were obtained by calculating the SERS intensity of two test lines for real food sample detection. Our proposed Mag@Au-ICA method has good potential to become a powerful POCT technology for illegal food additive monitoring because of its outstanding performance, simple operation, flexible mode, short detection time (30 min), high sensitivity (pg mL^−1^ level), good reproducibility, and specificity.

## Figures and Tables

**Table 1:** Comparison with other immunochromatographic methods.

Compound	Method	LOD (ng/mL)	Sample Types	Reference
CLE	Fluorescent ICA	0.16	Swine urine	2019 [49]
RAC	Colorimetric ICA	0.1	Animal feed	2019 [50]
RAC	Colorimetric ICA	2	Pork, pig feed	2020 [51]
CLE/RAC	Colorimetric ICA	0.2/0.12	Pork, mutton	2020 [42]
CLE	Colorimetric/SERS ICA	0.05	Pork, chicken, sausage	2021 [16]
CLE	Colorimetric ICA	1	Milk, tenderloin, pork	2021 [52]
CLE	Colorimetric ICA	0.13	Beef, pork liver	2022 [53]
CLE/RAC	Magnetic-SERS ICA	0.0078/0.0035	Pork, mutton, beef	This work

## Data Availability

Not applicable.

## Supplementary Materials

The following supporting information can be downloaded at: https://www.mdpi.com/article/10.3390/bios12090709/s1, Figure S1: UV-vis absorption spectra of Fe_3_O_4_ MNPs, Mag@Au seed NPs and Mag@Au MNPs; Figure S2: Effect of DTNB concentration on SERS intensity of Mag@Au tags; Figure S3: Raman peaks at 1331 cm^−1^ of Mag@Au after dispersion in distilled water containing 1% Tween at different pH (3–11) for 24 h; Figure S4: FTIR spectrum of antibody (red line), immuno-Mag@Au tag (blue line) and Mag@Au NPs (orange line); Figure S5: Effect of NC membranes with different pore sizes on competition inhibition rate; Figure S6: Effect of running buffer composition, reaction time, incubation time and enrichment system on competitive inhibition rate; Figure S7: Specificity results of Mag-ICA; Figure S8: Repeatability result of Mag-ICA; Table S1: the recoveries of CLE and RAC in actual food samples detected by the established Mag-ICA.
