# Supplementary material for: Magnetic Nanotag-Based Colorimetric/SERS Dual-Readout Immunochromatography for Ultrasensitive Detection of Clenbuterol Hydrochloride and Ractopamine in Food Samples
Source: Biosensors (Basel). 2022 Sep 1;12(9):709. doi: 10.3390/bios12090709 (PMC9496078; doi:10.3390/bios12090709)
Supplement: Supplementary file 1 [file biosensors-12-00709-s001.zip › biosensors-1878230-supplementary.pdf]

Supporting information

Magnetic Nanotag-Based Colorimetric/SERS Dual-Readout Immunochromatography for Ultrasensitive Detection of Clenbuterol hydrochloride and Ractopamine in food Samples

Ting Wu [1,2], Jiaxuan Li [1,2], Shuai Zheng [3], Qing Yu [3], Kezong Qi [1,3], Ying Shao [1,3], Chongwen Wang [1,2,3,*], Jian Tu [1,*] and Rui Xiao [2,*]

1 Anhui Province Key Laboratory of Veterinary Pathobiology and Disease Control, College of Animal Science and Technology, Anhui Agricultural University, Hefei 230036, China
2 Beijing Institute of Microbiology and Epidemiology, Beijing 100850, China
3 College of Life Sciences, Anhui Agricultural University, Hefei 230036, China
* Correspondence: wangchongwen1987@126.com (C.W.); tujian1980@126.com (J.T.); ruixiao203@sina.com (R.X.)

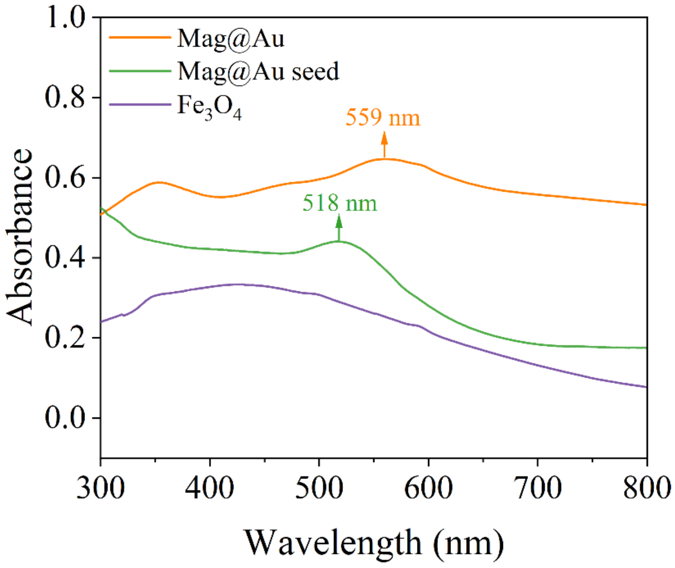

Figure S1 UV-vis absorption spectra of Fe_3O_4 MNPs, Mag@Au seed NPs and Mag@Au MNPs.

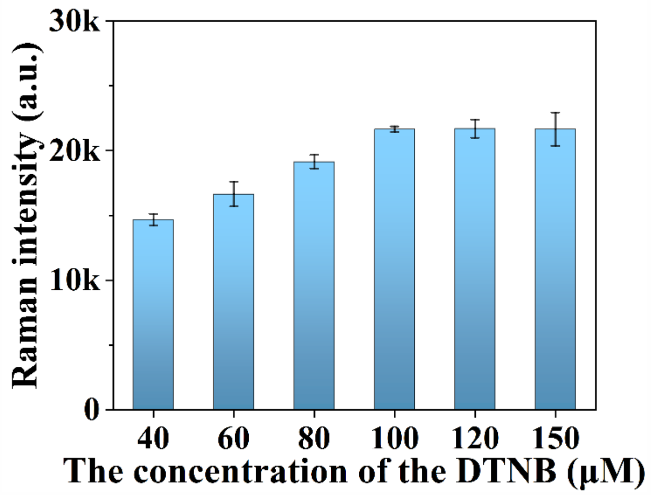

Figure S2 Effect of DTNB concentration on SERS intensity of Mag@Au tags.

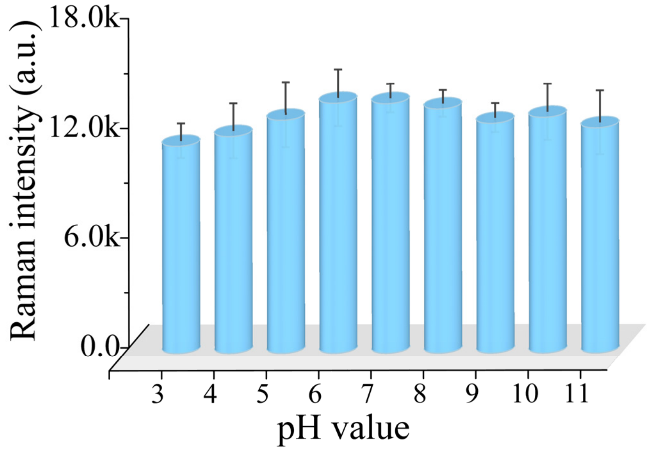

Figure S3 Raman peaks at 1331 cm^{-1} of Mag@Au after dispersion in distilled water containing 1% Tween at different pH (3-11) for 24 h.

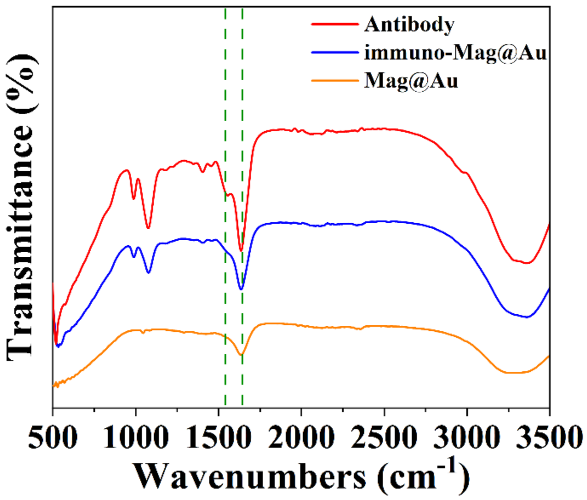

Figure S4 FTIR spectrum of antibody (red line), immuno-Mag@Au tag (blue line) and Mag@Au NPs (orange line). The characteristic absorption peaks corresponding to protein amide bands I (1641 cm^{-1}) and II (1530 cm^{-1} appearing in immuno-Mag@Au tags reveals the successful coupling of antibody.

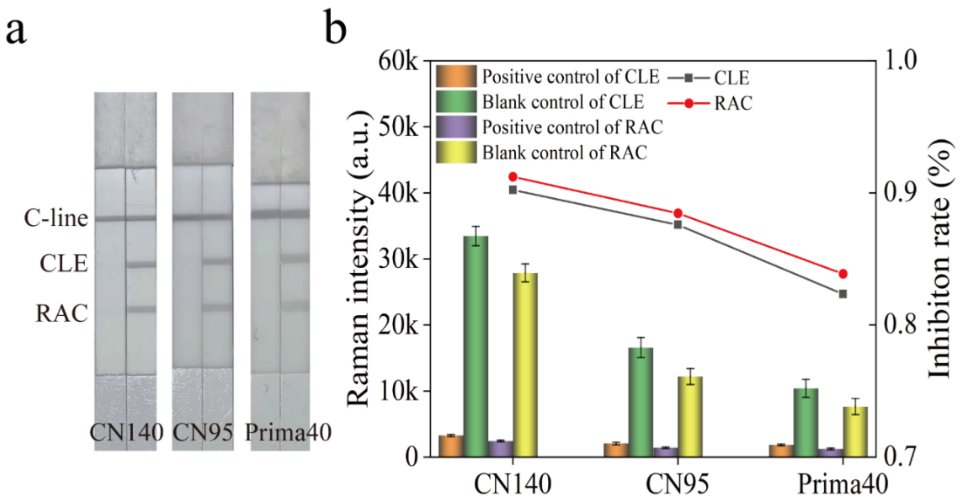

Figure S5 Effect of NC membranes with different pore sizes on competition inhibition rate. Diagrams of (a) test strips, (b) SERS signal intensity and inhibition rate. The error bar is the standard deviation of five independent tests based on Mag-ICA.

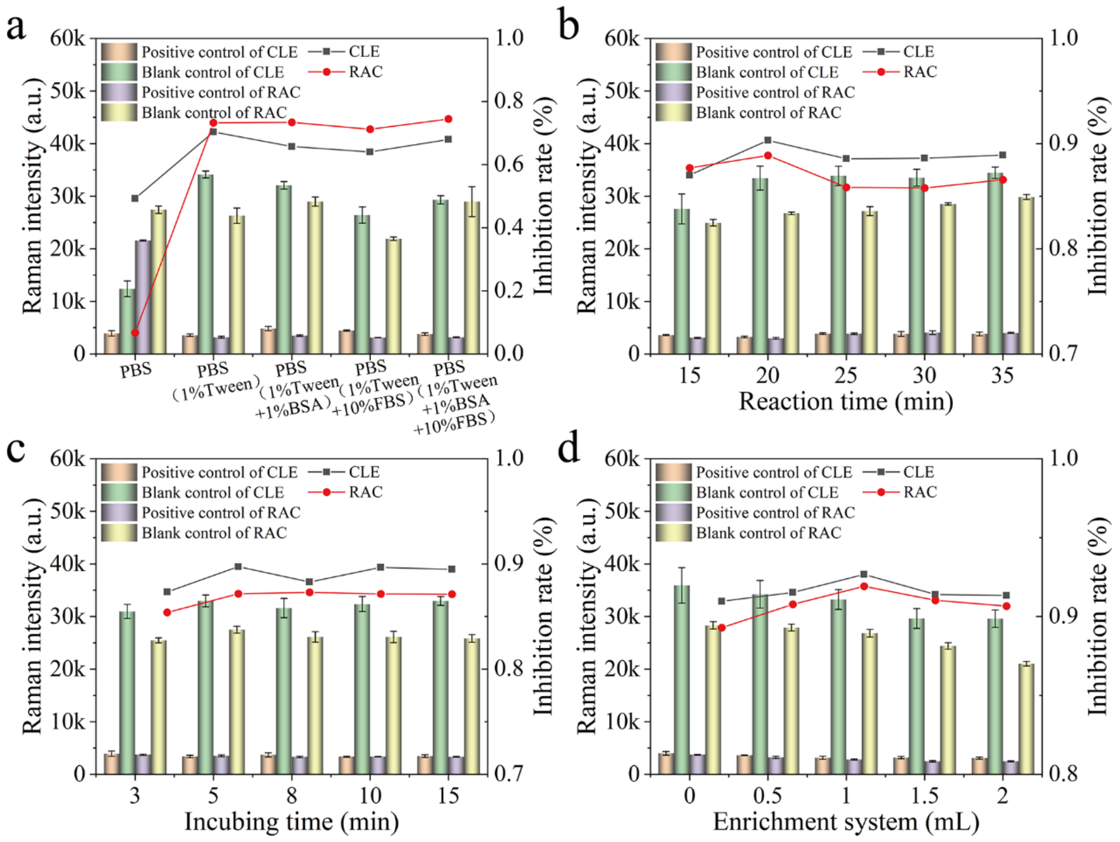

Figure S6 Effect of (a) running buffer composition, (b) reaction time, (c) incubation time and (d) enrichment system on competitive inhibition rate. The error bar is the standard deviation of five independent tests based on Mag-ICA.

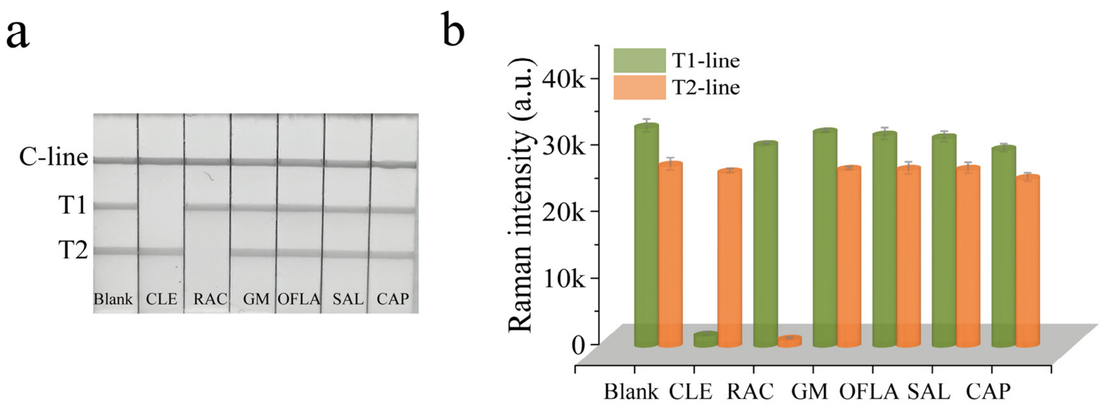

Figure S7 Specificity of Mag-ICA (a) Immunochromatographic test strip results in the presence of six foodborne hazards (CLE, RAC, GM, OFLA, SAL, and CAP), (b) corresponding test strip Raman signal values at 1331 cm^{-1}.

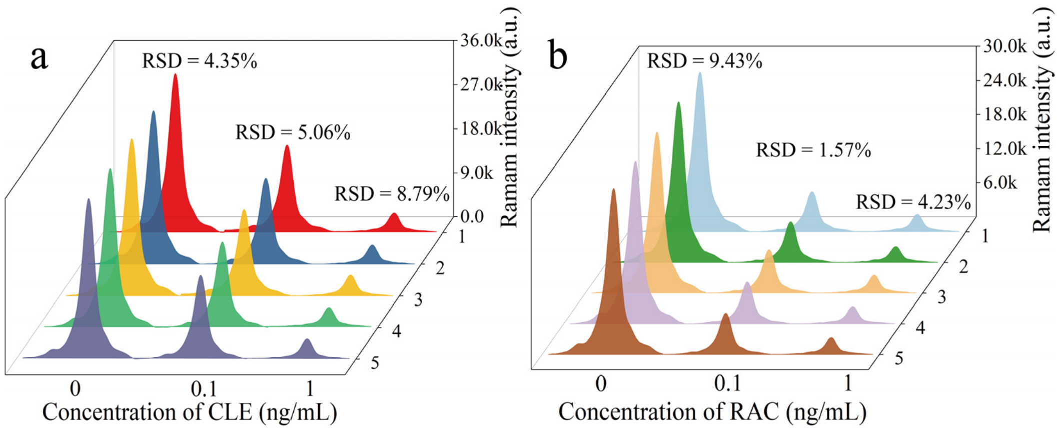

Figure S8 Raman peak diagram for five tests at (a) CLE and (b) RAC concentrations of 0 ng mL^{-1}, 0.1 ng mL^{-1} and 1 ng mL^{-1}.

Table S1. the recoveries of CLE and RAC in actual food samples detected by the established Mag-ICA.

**Samples**	**Added (ng mL^{-1})**		**Found (ng mL^{-1})**		**RSD (%)**		**Recovery ratio (%)**	
	**CLE**	**RAC**	**CLE**	**RAC**	**CLE**	**RAC**	**CLE**	**RAC**
**Pork**	0.01	0.01	0.0109	0.0111	8.45	4.87	108.67	110.60
	0.1	0.1	0.0929	0.1000	4.33	4.63	92.87	100.05
	1	1	0.9323	1.0177	1.99	1.44	93.23	101.77
**Beef**	0.01	0.01	0.0101	0.0090	2.33	1.40	101.36	89.91
	0.1	0.1	0.0964	0.1098	6.58	4.80	96.40	109.77
	1	1	0.9007	0.8963	7.18	9.96	90.07	89.63
**Mutton**	0.01	0.01	0.0107	0.0107	3.21	6.50	107.49	106.80
	0.1	0.1	0.1029	0.1051	6.09	8.39	102.93	105.14
	1	1	0.9442	1.0834	1.56	1.98	94.42	108.34

References

1. Wang, J.; Wu, X.; Wang, C.; Shao, N.; Dong, P.; Xiao, R.; Wang, S., Magnetically Assisted Surface-Enhanced Raman Spectroscopy for the Detection of Staphylococcus aureus Based on Aptamer Recognition. *ACS Appl Mater Interfaces* **2015,** *7* (37), 20919-29.

2. Zheng, S.; Wu, T.; Li, J.; Jin, Q.; Xiao, R.; Wang, S.; Wang, C., Difunctional immunochromatographic assay based on magnetic quantum dot for ultrasensitive and simultaneous detection of multiple mycotoxins in foods. *Sens Actuators B Chem* **2022,** *359*, 131528.
